# Supplementary material for: The neurotransmitter calcitonin gene-related peptide shapes an immunosuppressive microenvironment in medullary thyroid cancer
Source: Nat Commun. 2024 Jul 19;15:5555. doi: 10.1038/s41467-024-49824-7 (PMC11271530; doi:10.1038/s41467-024-49824-7)
Supplement: Supplementary file 1 — Supplementary Information [file 41467_2024_49824_MOESM1_ESM.pdf]

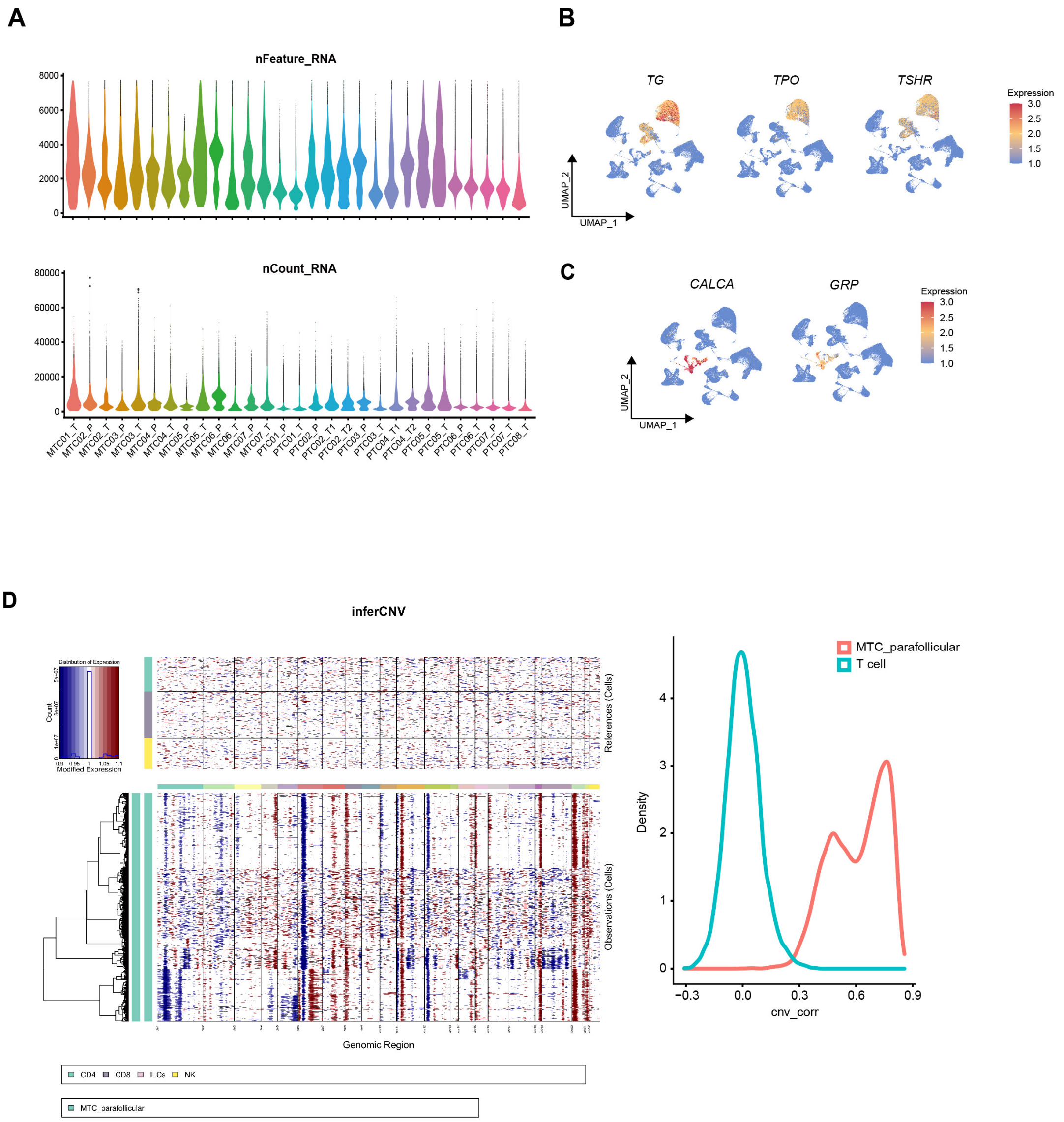
A
nFeature_RNA
nCount_RNA
MTC01_T
MTC02_P
MTC02_T
MTC03_P
MTC03_T
MTC04_P
MTC04_T
MTC05_P
MTC05_T
MTC06_P
MTC06_T
MTC07_P
MTC07_T
PTC01_P
PTC01_T
PTC02_P
PTC02_T1
PTC02_T2
PTC03_P
PTC03_T
PTC04_T1
PTC04_T2
PTC05_P
PTC05_T
PTC06_P
PTC06_T
PTC07_P
PTC07_T
PTC08_T
B
TG
TPO
TSHR
Expression
UMAP_1
UMAP_2
C
CALCA
GRP
Expression
UMAP_1
UMAP_2
D
inferCNV
Distribution of Expression
Count
Modified Expression
References (Cells)
Observations (Cells)
Genomic Region
CD4
CD8
ILCs
NK
MTC_parafollicular
MTC_parafollicular
T cell
Density
cnv_corr

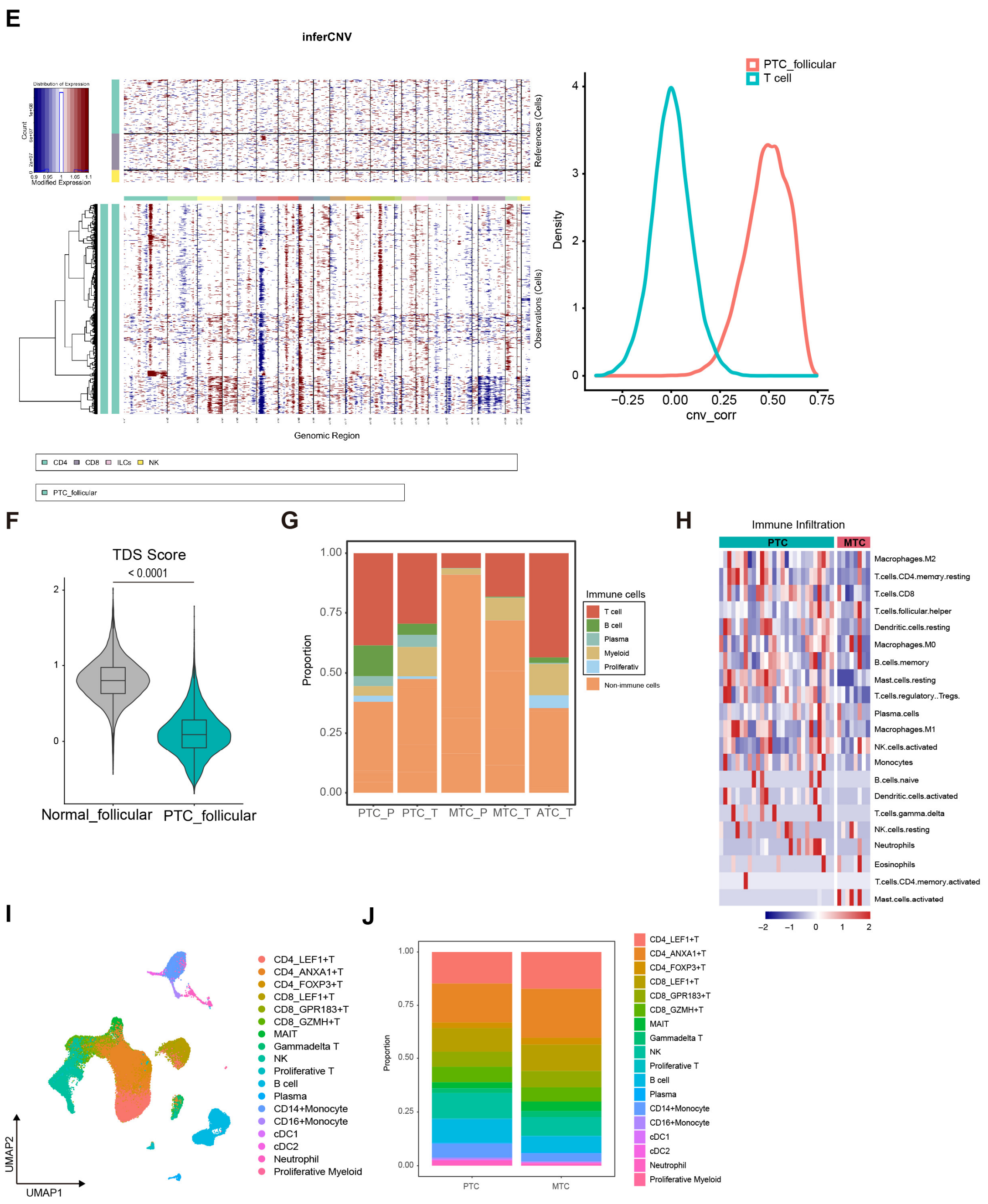

Supplementary Figure 1. Human thyroid cancer single-cell cell landscape revealed low immune infiltration in MTC, related to Figure 1. (A) Violin plot showing the gene signature and the count information for each sample. **(B)** Thyroid functional genes expression was plotted on feature plots to mark follicular epithelial cells. **(C)** The expression of *CALCA* and *GRP* was presented on feature plots to mark parafollicular cell. **(D)** The copy number alterations of malignant MTC cells inferred by inferCNV. T cells were selected as normal reference, and the copy number gain was shown in red

color and the copy number loss were shown in blue color, similarly hereinafter. Density plot showed the correlation distribution of CNV signal among reference cells and MTC tumor cells. **(E)** The copy number alterations of PTC tumor cells inferred by inferCNV. T cells were selected as reference. Density plot showed the correlation distribution of CNV signal among reference cells and PTC tumor cells. **(F)** The Thyroid differentiation score (TDS) was calculated in normal follicular epithelial cells and in PTC tumor cells. The box inside illustrates the interquartile range in relation to the median, while the middle lines represent the median, and the lower and upper hinges denote the 25-75% interquartile range (IQR), with whiskers extending up to a maximum of 1.5 times IQR. Calculated by two-sided Wilcoxon rank-sum test. **(G)** Cell type proportions of immune cell and non-immune cells. Each stacked bar represents tumor or adjacent normal tissue of PTC, MTC and ATC. MTC_T, PTC_T and ATC_T represents tumors of MTC, PTC and ATC, respectively. MTC_P and PTC_P represents peripheral thyroid tissue of MTC**.** **(H)** In the bulk-RNA data, immune cell types inferred by CIBERSORT in PTC and MTC are shown. **(I)** UMAP visualization of PBMC cells, colored by cell type annotations. **(J)** Bar graph showing the proportions of cell type in PBMC from PTC or MTC.

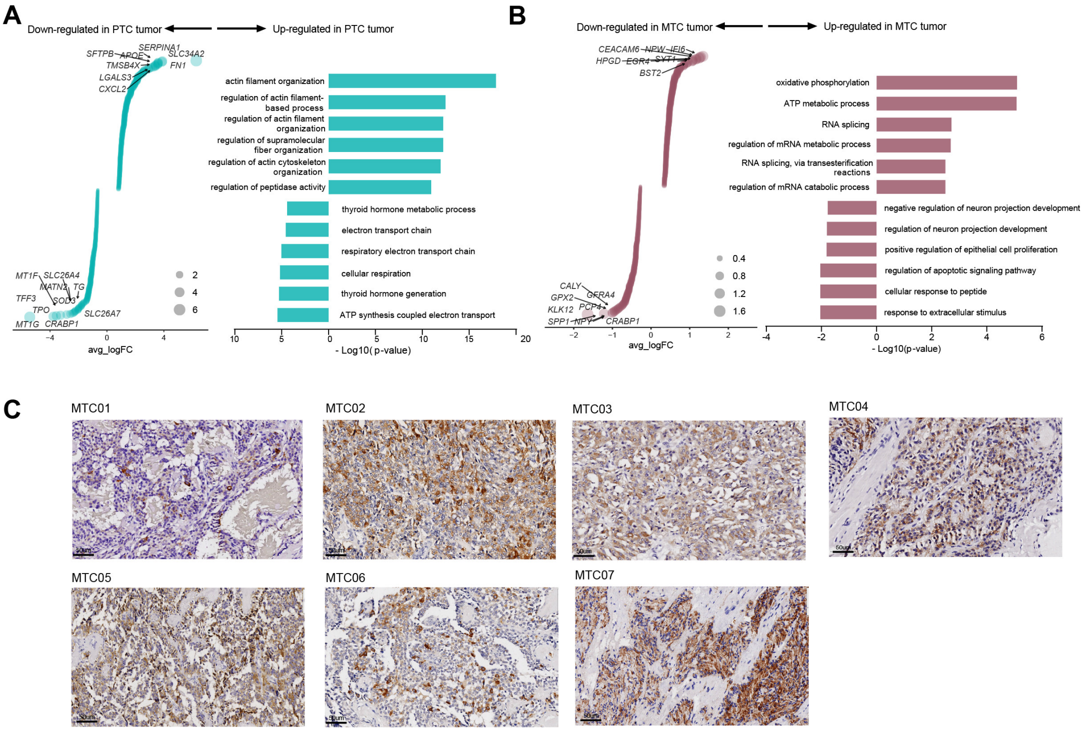

A
Down-regulated in PTC tumor
Up-regulated in PTC tumor
actin filament organization
regulation of actin filament-based process
regulation of actin filament organization
regulation of supramolecular fiber organization
regulation of actin cytoskeleton organization
regulation of peptidase activity
thyroid hormone metabolic process
electron transport chain
respiratory electron transport chain
cellular respiration
thyroid hormone generation
ATP synthesis coupled electron transport
avg_logFC
- Log10(p-value)
B
Down-regulated in MTC tumor
Up-regulated in MTC tumor
oxidative phosphorylation
ATP metabolic process
RNA splicing
regulation of mRNA metabolic process
RNA splicing, via transesterification reactions
regulation of mRNA catabolic process
negative regulation of neuron projection development
regulation of neuron projection development
positive regulation of epithelial cell proliferation
regulation of apoptotic signaling pathway
cellular response to peptide
response to extracellular stimulus
avg_logFC
- Log10(p-value)
C
MTC01
MTC02
MTC03
MTC04
MTC05
MTC06
MTC07

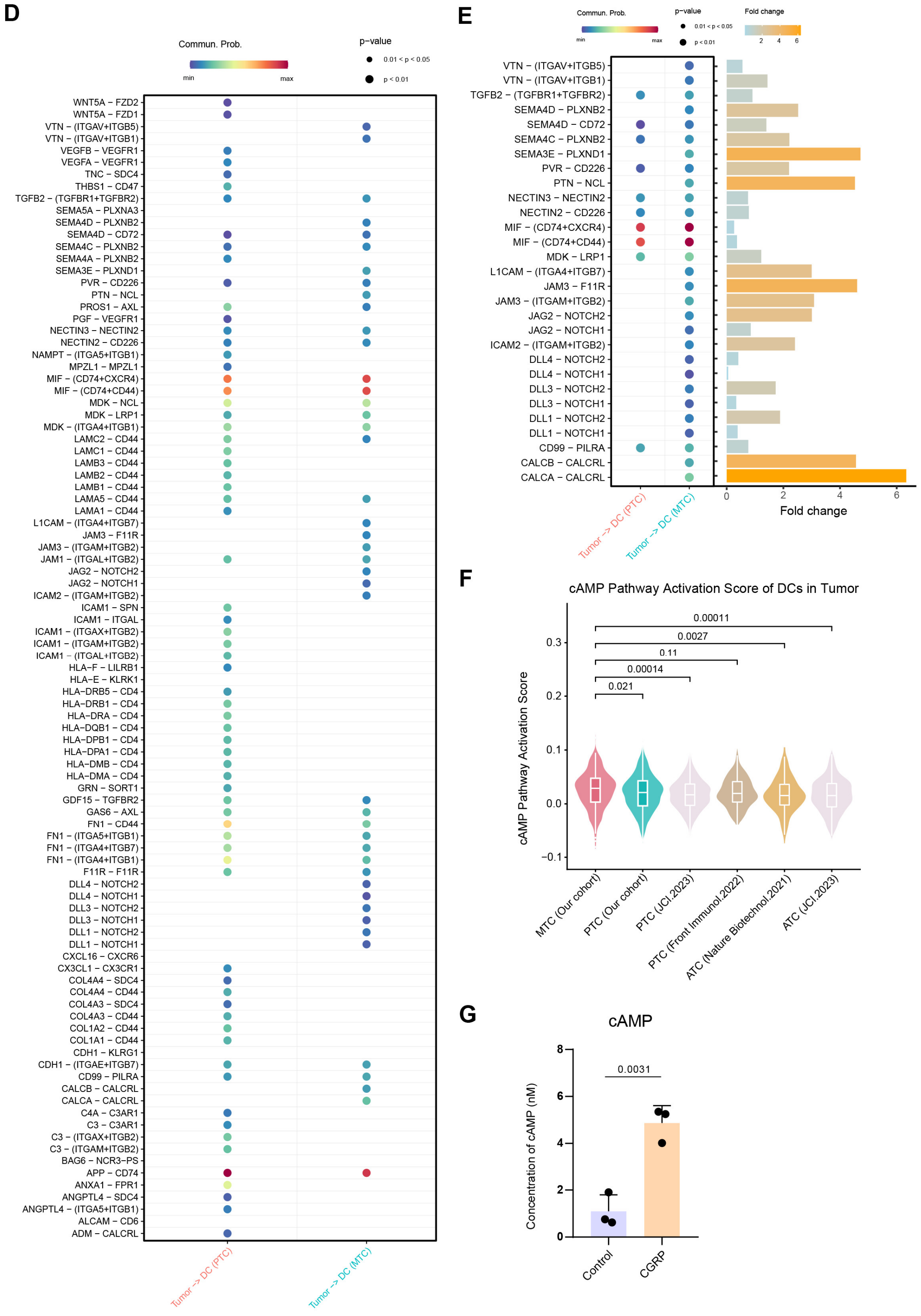

Supplementary Figure 2. Tumor-specific CGRP interacts with DCs in MTC, related to Figure 2. (A) Differentially expressed genes and enrichment pathways in comparison of PTC tumor cells and normal follicular cells were shown. Dot size in left

panel represented the average Log2 (fold change) of differentially expressed genes, respectively. **(B)** Differentially expressed genes and enrichment pathways in comparison of MTC tumor cells and normal parafollicular cells were shown. Dot size in left panel represented the average Log2 (fold change) of differentially expressed genes, respectively. **(C)** In the tumor region of MTC patients for single-cell sequencing (n=7), IHC staining for CGRP was shown. **(D)** Complete cellchat interaction plot between tumor cell and DCs was shown. **(E)** A dot plot was used to illustrate the upregulated receptor-ligand pairs in MTC compared to PTC, with the fold change on the right indicating the ratio of differences. **(F)** Violin plot showing the genes signature score of cAMP related pathways activation in DCs derived from tumor of MTC, PTC and public dataset of PTC and ATC. The box inside illustrates the interquartile range in relation to the median, while the middle lines represent the median, and the lower and upper hinges denote the 25-75% interquartile range (IQR), with whiskers extending up to a maximum of 1.5 times IQR. Calculated by two-sided Wilcoxon rank-sum test. **(G)** ELISA assay showed the concentration of cAMP (nM) after CGRP treatment (n=3 for each group). *P*-values from two-tailed Student's t test were represented. The experiments were performed independently for three times. Source data are provided as a Source Data file.

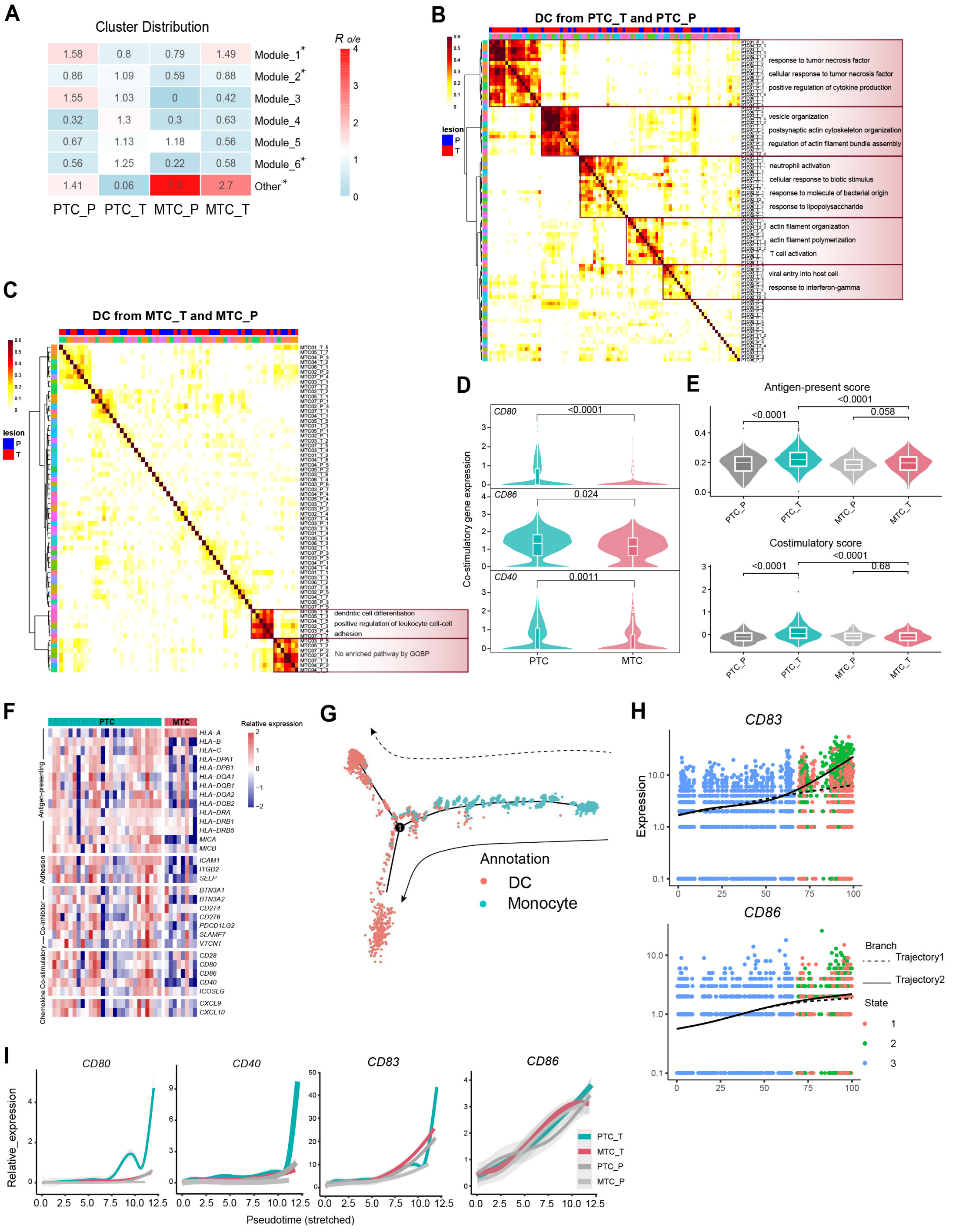
A
Cluster Distribution
1.58 0.8 0.79 1.49 Module_1*
0.86 1.09 0.59 0.88 Module_2*
1.55 1.03 0 0.42 Module_3
0.32 1.3 0.3 0.63 Module_4
0.67 1.13 1.18 0.56 Module_5
0.56 1.25 0.22 0.58 Module_6*
1.41 0.06 7.1 2.7 Other*
PTC_P PTC_T MTC_P MTC_T
R o/e
B
DC from PTC_T and PTC_P
lesion
P
T
response to tumor necrosis factor
cellular response to tumor necrosis factor
positive regulation of cytokine production
vesicle organization
postsynaptic actin cytoskeleton organization
regulation of actin filament bundle assembly
neutrophil activation
cellular response to biotic stimulus
response to molecule of bacterial origin
response to lipopolysaccharide
actin filament organization
actin filament polymerization
T cell activation
viral entry into host cell
response to interferon-gamma
C
DC from MTC_T and MTC_P
lesion
P
T
dendritic cell differentiation
positive regulation of leukocyte cell-cell adhesion
No enriched pathway by GOBP
D
Co-stimulatory gene expression
CD80 <0.0001
CD86 0.024
CD40 0.0011
PTC
MTC
E
Antigen-present score
<0.0001
<0.0001
0.058
Costimulatory score
<0.0001
<0.0001
0.68
PTC_P
PTC_T
MTC_P
MTC_T
F
PTC
MTC
Relative expression
Antigen-presenting
Adhesion
Co-inhibitor
Co-stimulatory
Chemokine
HLA-A
HLA-B
HLA-C
HLA-DPA1
HLA-DPB1
HLA-DQA1
HLA-DQB1
HLA-DQA2
HLA-DQB2
HLA-DRA
HLA-DRB1
HLA-DRB5
MICA
MICB
ICAM1
ITGB2
SELP
BTN3A1
BTN3A2
CD274
CD276
PDCD1LG2
SLAMF7
VTCN1
CD28
CD80
CD86
CD40
ICOSLG
CXCL9
CXCL10
G
Annotation
DC
Monocyte
H
CD83
Expression
CD86
Branch
Trajectory1
Trajectory2
State
1
2
3
I
CD80
CD40
CD83
CD86
Relative_expression
Pseudotime (stretched)
PTC_T
MTC_T
PTC_P
MTC_P

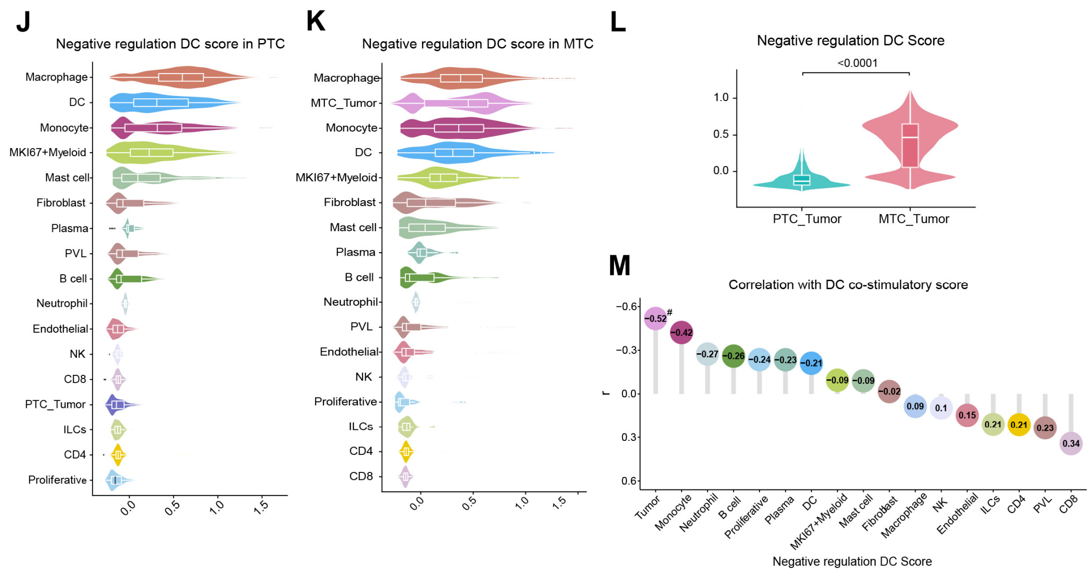

Supplementary Figure 3. DCs in MTC were dysfunctional and displayed distinct developmental trajectory compared to DCs in PTC, related to Figure 3. (A) Heatmap of the distribution ratio of modules in tumor and normal tissues of PTC and MTC, **p*<0.01. **(B)** Heatmap showing expression correlation of gene programs in DCs across tumor and normal tissue samples from PTC patient only. **(C)** Heatmap showing expression correlation of gene programs in DCs across tumor and normal tissue samples from MTC patient only. Unlike DCs in PTC, most of DCs in MTC did not show specific expression patterns. **(D)** The expression of co-stimulatory genes *CD80*, *CD86* and *CD40* between PTC and MTC was shown by violin plot. Calculated by two-sided Wilcoxon rank-sum test. **(E)** The gene scores of antigen-presenting, co-stimulatory signatures were calculated in DCs from different tissue origins and shown in violin plots. Calculated by two-sided Wilcoxon rank-sum test. **(F)** The expression of DC functional gene signatures was calculated from the bulk-RNA data and shown in heatmap. **(G)** The trajectory was colored according to the annotation of monocytes and DCs. **(H)** Pseudo-time expression of *CD83* and *CD86* along trajectories 1 and 2. **(I)** Plots showing *CD80*, *CD40*, *CD83* and *CD86* genes expression in tumor or peripheral normal tissue of PTC and MTC along trajectories. **(J)** Violin plot showed negative regulation of DC score in all cell types in PTC tumor. **(K)** Violin plot showed negative regulation of DC score in all cell types in MTC tumor. **(L)** Negative regulation of DC

score in MTC tumor cells and PTC tumor cells were displayed by violin plot. Calculated by two-sided Wilcoxon rank-sum test. **(M)** Correlation relationship between negative regulation DC score of all cell types and DCs' co-stimulatory score at sample level was showed by lollipop plot. r means correlation coefficient calculated by Pearson's correlation test, $^{\#}p<0.05$. The box inside violin plot in this Figure illustrate the interquartile range in relation to the median, while the middle lines represent the median, and the lower and upper hinges denote the 25-75% interquartile range (IQR), with whiskers extending up to a maximum of 1.5 times IQR.

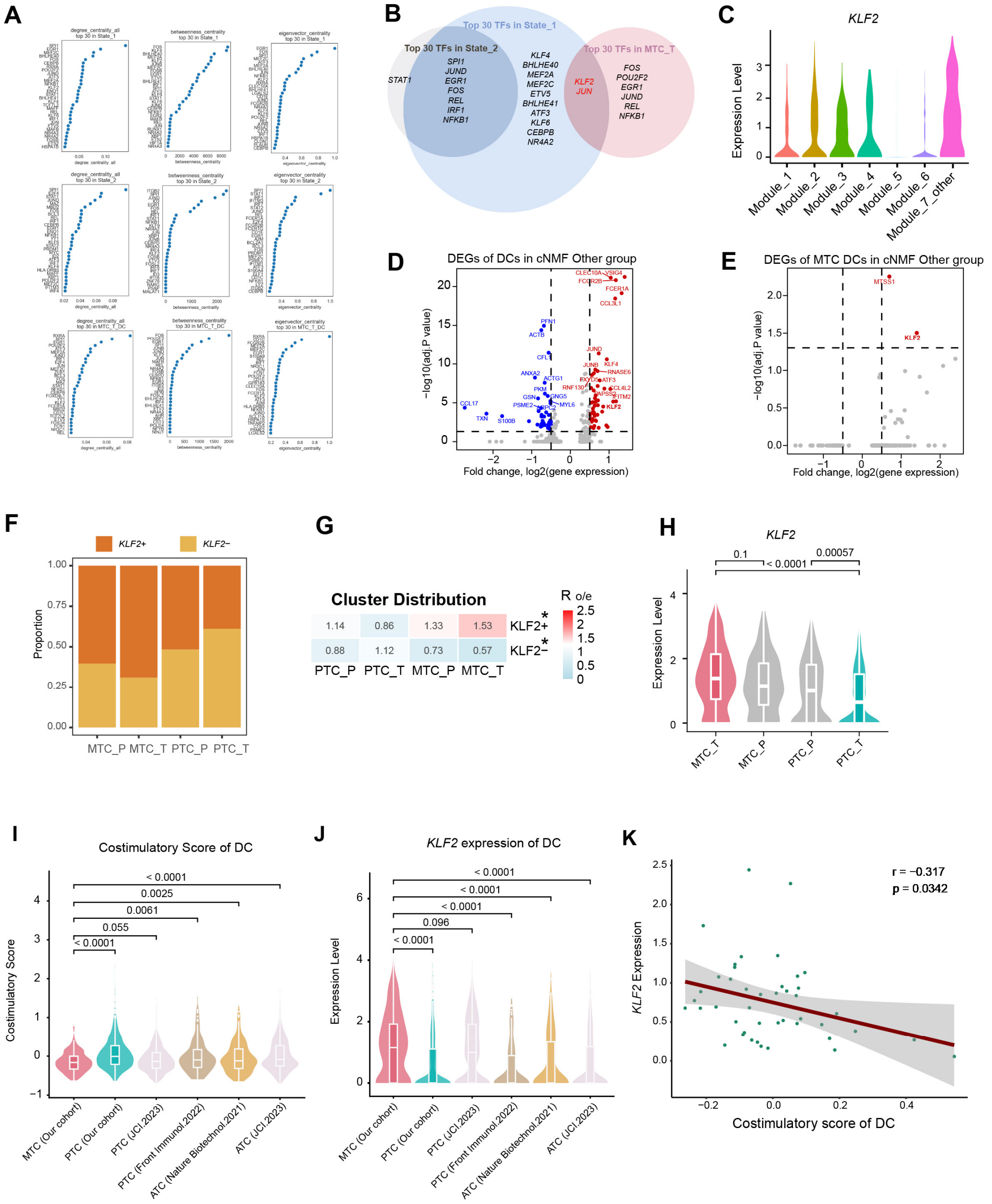

Supplementary Figure 4. Elevating level of transcription factor KLF2 contributed to the development of intra-tumoral DCs in MTC, related to Figure 4. (A) Top 30 transcription factor of degree centrality, betweenness centrality and eigenvector centrality in State1, State2 and MTC_T groups. **(B)** The key transcription factors in groups State1 and State2, as well as in MTC tumors, were identified based on overlapped transcription factors of degree centrality, betweenness centrality and eigenvector centrality. The final selection of key transcription factors required them to

meet the following criteria: 1) Specific to State1 and without a significant role in State2; 2) Considered to be key transcription factors in MTC tumor. **(C)** Violin plot showing the expression of *KLF2* in DCs from different cNMF modules. **(D)** Volcano plot showing the DEGs of the "Other" group compared to cNMF module 1-6. Genes in red color are upregulated in "Other" group in and genes in blue color are downregulated. **(E)** Volcano plot showing the DEGs of MTC DCs in the "Other" group recognized by cNMF analysis. **(F)** Cell type proportions of *KLF2*$^{+}$ and *KLF2*$^{-}$ DCs in tumors or normal tissues of MTC and PTC. Here, PTC_T and MTC_T represent tumors of PTC and MTC respectively, as above. PTC_P represents normal thyroid tissue of PTC and MTC_P represents normal thyroid tissue of MTC. **(G)** Heatmap of the distribution ratio of *KLF2*$^{+}$ and *KLF2*$^{-}$ DCs in the tumor or in normal tissue of MTC and PTC. Calculated by Chi-square test, **p*<0.01. **(H)** Violin plot showing the expression of *KLF2* in DCs derived from tumor or normal tissues of PTC and MTC. Calculated by two-sided Wilcoxon rank-sum test. **(I)** Violin plot showing the genes signature score of Co-stimulation in DCs derived from tumor of MTC, PTC and public dataset of PTC and ATC. Calculated by two-sided Wilcoxon rank-sum test. **(J)** Violin plot showing the genes expression of *KLF2* in DCs derived from tumor of MTC, PTC and public dataset of PTC and ATC. Calculated by two-sided Wilcoxon rank-sum test. **(K)** Scatter plot showing the correlation relationship between *KLF2* expression and co-stimulatory score of tumor DCs at the sample level (n=45). r indicates the correlation coefficient calculated by Spearman correlation test. The box inside violin plot in this Figure illustrate the interquartile range in relation to the median, while the middle lines represent the median, and the lower and upper hinges denote the 25-75% interquartile range (IQR), with whiskers extending up to a maximum of 1.5 times IQR. Source data are provided as a Source Data file.

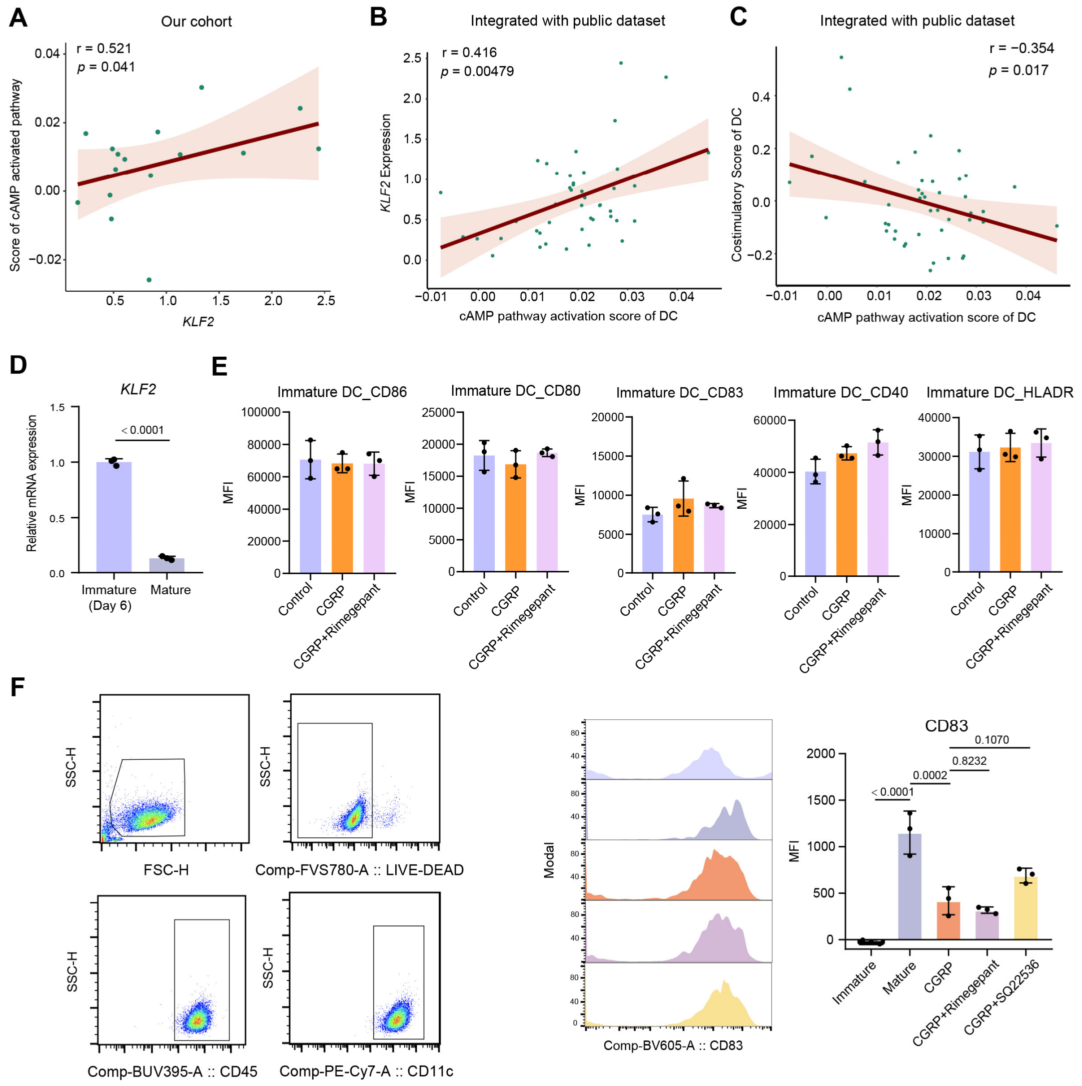

Supplementary Figure 5. CGRP drove the development of dysfunctional DCs by preventing the loss of KLF2, related to Figure 5. (A) Scatter plot showing the correlation relationship between *KLF2* expression and cAMP activation level of tumor DCs in our cohort (n=16). r means correlation coefficient calculated by Spearman's correlation test. **(B)** Scatter plot showing the correlation relationship between *KLF2* expression and cAMP activation level of tumor DCs when integrated with public single-cell dataset (n=45). **(C)** Scatter plot showing the correlation relationship between DC co-stimulatory score and cAMP activation level of tumor DCs when integrated with public single-cell dataset (n=45). **(D)** Relative mRNA expression of *KLF2* in immature and mature DCs of the control group after cytokine cocktail stimulation (n=3 for each

group). The was presented as mean ± SD. *P* value between groups was calculated by student's t-test. **(E)** Mean fluorescence intensity (MFI) of functional markers on immature DCs (n=3 for each group). The was presented as mean ± SD. **(F)** Representative flow cytometry histogram and mean fluorescence intensity (MFI) of CD83 expression on mature DCs (n=3 for each group). The was presented as mean ± SD. *P* value was calculated by one-way ANNOVAR compared to control group. The experiments were performed independently for three times. Source data are provided as a Source Data file.

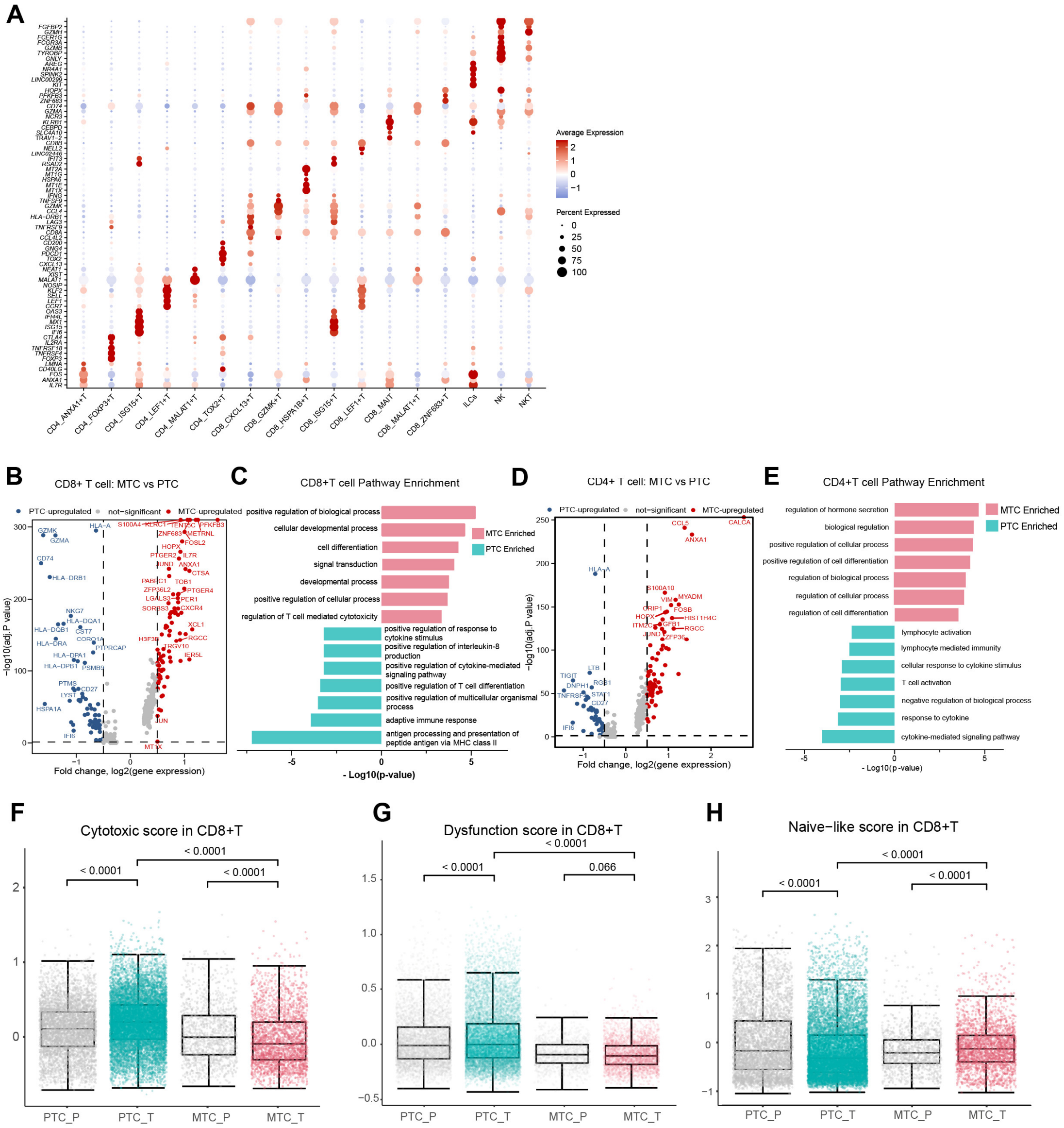
A
Average Expression
Percent Expressed
CD4_ANXA1+T
CD4_FOXP3+T
CD4_ISG15+T
CD4_LEF1+T
CD4_MALAT1+T
CD4_TOX2+T
CD8_CXCL13+T
CD8_GZMK+T
CD8_HSPA1B+T
CD8_ISG15+T
CD8_LEF1+T
CD8_MAIT
CD8_MALAT1+T
CD8_ZNF683+T
ILCs
NK
NKT
B
CD8+ T cell: MTC vs PTC
PTC-upregulated
not-significant
MTC-upregulated
−log10(adj.P value)
Fold change, log2(gene expression)
C
CD8+T cell Pathway Enrichment
MTC Enriched
PTC Enriched
positive regulation of biological process
cellular developmental process
cell differentiation
signal transduction
developmental process
positive regulation of cellular process
regulation of T cell mediated cytotoxicity
positive regulation of response to cytokine stimulus
positive regulation of interleukin-8 production
positive regulation of cytokine-mediated signaling pathway
positive regulation of T cell differentiation
positive regulation of multicellular organismal process
adaptive immune response
antigen processing and presentation of peptide antigen via MHC class II
- Log10(p-value)
D
CD4+ T cell: MTC vs PTC
PTC-upregulated
not-significant
MTC-upregulated
−log10(adj.P value)
Fold change, log2(gene expression)
E
CD4+T cell Pathway Enrichment
MTC Enriched
PTC Enriched
regulation of hormone secretion
biological regulation
positive regulation of cellular process
positive regulation of cell differentiation
regulation of biological process
regulation of cellular process
regulation of cell differentiation
lymphocyte activation
lymphocyte mediated immunity
cellular response to cytokine stimulus
T cell activation
negative regulation of biological process
response to cytokine
cytokine-mediated signaling pathway
- Log10(p -value)
F
Cytotoxic score in CD8+T
< 0.0001
< 0.0001
< 0.0001
PTC_P
PTC_T
MTC_P
MTC_T
G
Dysfunction score in CD8+T
< 0.0001
< 0.0001
0.066
PTC_P
PTC_T
MTC_P
MTC_T
H
Naive−like score in CD8+T
< 0.0001
< 0.0001
< 0.0001
PTC_P
PTC_T
MTC_P
MTC_T

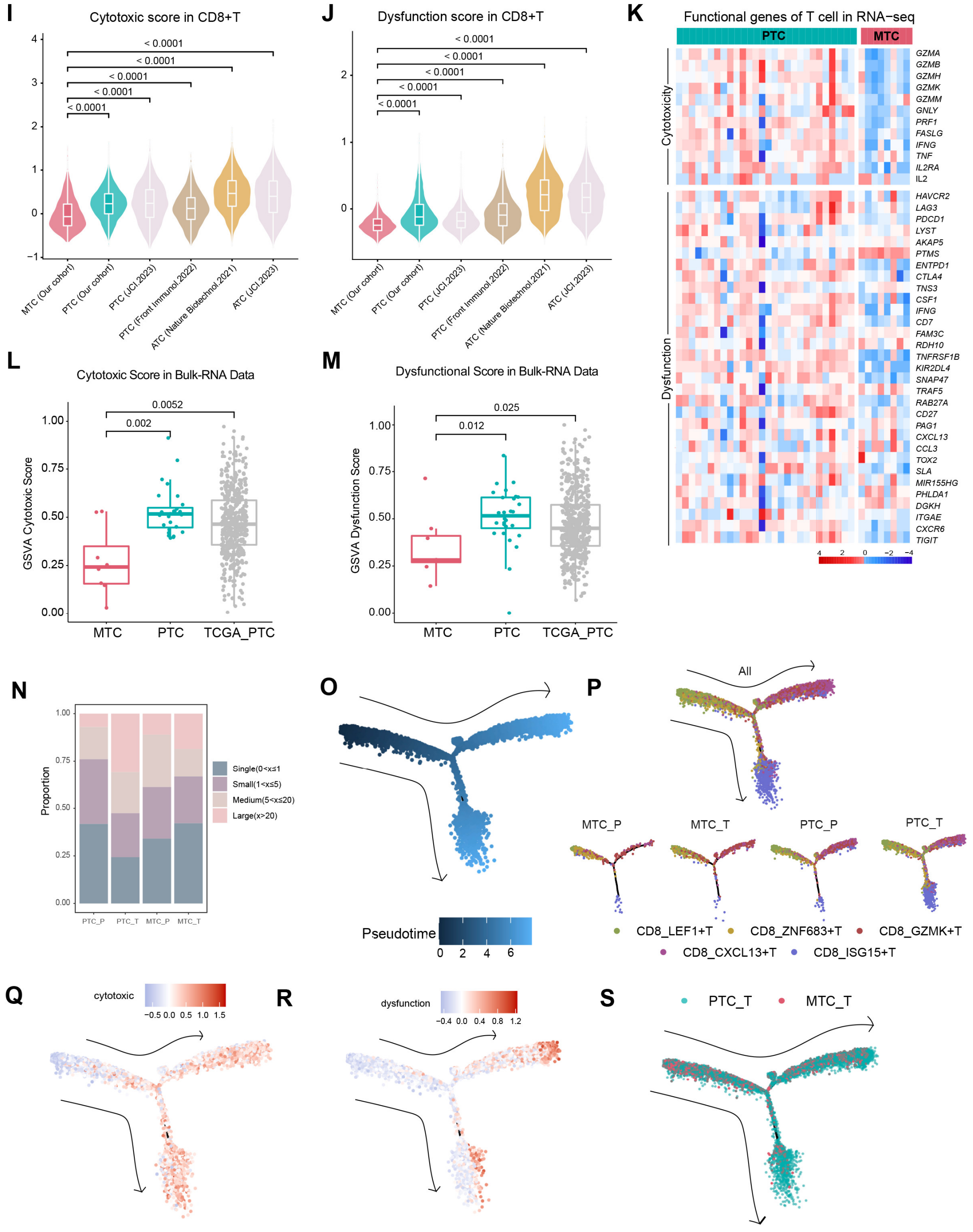

Supplementary Figure 6. Dysfunctional DCs attenuated the activation of CD8^{+} T cells in MTC, related to Figure 6. (A) Dot plot showing top marker genes of T cell subpopulations. **(B)** The DEGs of CD8^{+} T cells between PTC and MTC were shown in the volcano plot. **(C)** Pathways that are enriched for CD8^{+} T cells in PTC and MTC. **(D)** The DEGs of CD4^{+} T cells between PTC and MTC were shown in the volcano plot. **(E)**

Pathways that are enriched for $CD4^+$ T cells in PTC and MTC. **(F-H)** Box plots showing the expression scores of naive-like, cytotoxic and dysfunctional gene signatures among $CD8^+$ T cells in PTC and MTC tumors and adjacent tissues, respectively. The boxplot illustrates the interquartile range in relation to the median, while the middle lines represent the median, and the lower and upper hinges denote the 25-75% interquartile range (IQR), with whiskers extending up to a maximum of 1.5 times IQR. Here, PTC_T and MTC_T represent tumors of PTC and MTC respectively, as above. PTC_P represents normal thyroid tissue of PTC and MTC_P represents normal thyroid tissue of MTC. **(I-J)** Violin plot showing the genes signature score of the cytotoxicity and dysfunction in tumor $CD8^+$ T derived from MTC, PTC and public dataset of PTC and ATC. The box inside the violin plot illustrates the interquartile range in relation to the median, while the middle lines represent the median, and the lower and upper hinges denote the 25-75% interquartile range (IQR), with whiskers extending up to a maximum of 1.5 times IQR. Calculated by two-sided Wilcoxon test. **(K)** Heatmap showing the expression of T cell functional genes in PTC and MTC in bulk-RNA data. **(L-M)** The expression scores of the cytotoxic and dysfunctional gene signatures in PTC (28 samples from our cohort and 502 samples from TCGA public dataset) and 8 samples MTC in the bulk-RNA data. The box plot illustrates the interquartile range in relation to the median, while the middle lines represent the median, and the lower and upper hinges denote the 25-75% interquartile range (IQR), with whiskers extending up to a maximum of 1.5 times IQR. *P*-value was determined using two-sided Wilcoxon rank-sum test. **(N)** Bar graph showing the distribution of TCR expansion in the tumor or in the adjacent tissue of PTC and MTC. **(O)** The developmental trajectory of $CD8^+$ T cells as inferred by Monocle2. **(P)**The trajectories of $CD8^+$ T cells were segmented by tissue origin and colored according to subpopulations. **(Q-R)** The trajectory of $CD8^+$ T cells was colored based on the expression level of cytotoxic and dysfunctional gene signatures. **(S)** The trajectory of tumor $CD8^+$ T cells was colored according to PTC and MTC origin. Source data are provided as a Source Data file.

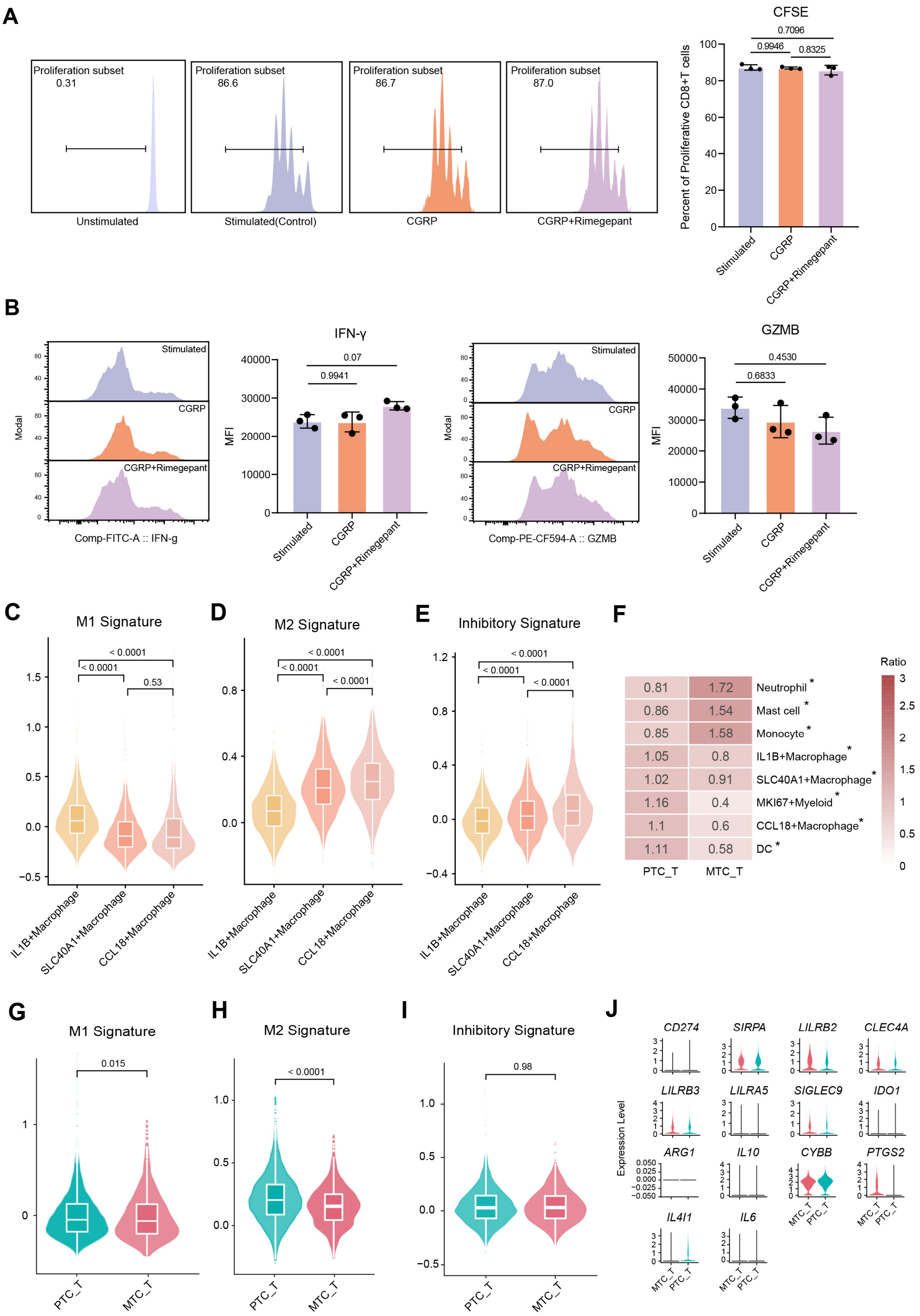

	PTC_T	MTC_T
Neutrophil*	0.81	1.72
Mast cell*	0.86	1.54
Monocyte*	0.85	1.58
IL1B+Macrophage*	1.05	0.8
SLC40A1+Macrophage*	1.02	0.91
MKI67+Myeloid*	1.16	0.4
CCL18+Macrophage*	1.1	0.6
DC*	1.11	0.58

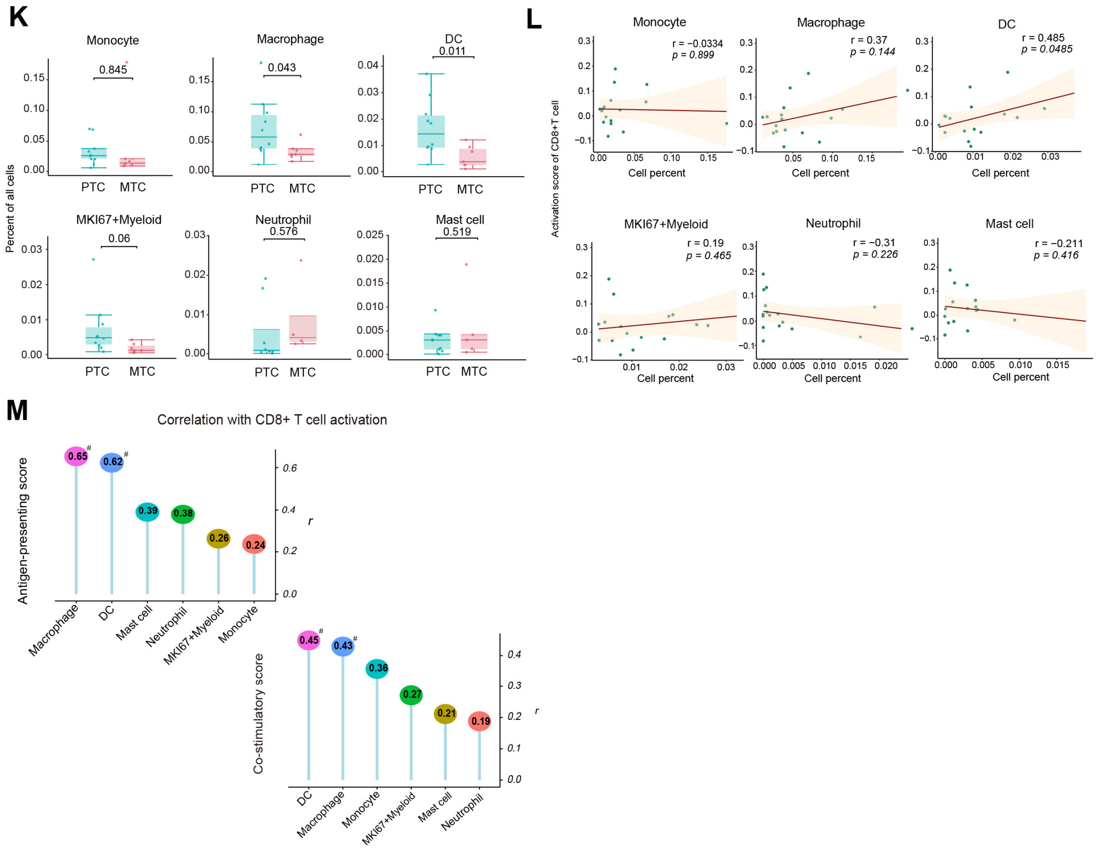

Supplementary Figure 7. Dysfunctional DCs influenced by CGRP responsible for inducing the suppressive characteristics of CD8^{+} T cells in MTC, related to Figure 7. **(A)** Percentage of proliferating CD8^{+} T cells was shown under the treatment of CGRP (n=3 for each group). The data was presented as mean ± SD. *P* values between groups were calculated by one-way ANNOVAR. **(B)** MFI of IFN-γ and GZMB expression on mature CD8^{+} T cells (n=3 for each group). The data was presented as mean ± SD. *P* value was calculated by one-way ANNOVAR compared to control group. The experiments were performed independently for three times. **(C-E)** Violin plot showing the genes signature score of M1, M2 and immune inhibition in macrophage subtypes. The box inside the violin plot illustrates the interquartile range in relation to the median, while the middle lines represent the median, and the lower and upper hinges denote the 25–75% interquartile range (IQR), with whiskers extending up to a maximum of 1.5 times IQR. Calculated by two-sided Wilcoxon test. **(F)** Heatmap of the distribution ratio of myeloid cell subtypes in MTC and PTC tumor, *p<0.01. **(G-I)** Violin plot showing

the genes signature score of M1, M2 and immune inhibition in tumor macrophages derived from MTC and PTC. The box inside the violin plot illustrates the interquartile range in relation to the median, while the middle lines represent the median, and the lower and upper hinges denote the 25-75% interquartile range (IQR), with whiskers extending up to a maximum of 1.5 times IQR. Calculated by two-sided Wilcoxon test. **(J)** Violin plot showing the gene expression of immune inhibition in tumor macrophages derived from MTC and PTC. **(K)** Box plot presented the proportions of sub-populations of myeloid cells. The boxplot illustrates the interquartile range in relation to the median, while the middle lines represent the median, and the lower and upper hinges denote the 25-75% interquartile range (IQR), with whiskers extending up to a maximum of 1.5 times IQR. Calculated by two-sided Wilcoxon test. **(L)** Scatter plot showed the correlation relationship between the proportion of myeloid sub-populations and the activation score of $CD8^{+}$T cell at sample level. r means the correlation coefficient calculated by Pearson's correlation test. **(M)** Lollipop plot showed the correlation relationship between co-stimulatory score or antigen-presenting score with the activation score of $CD8^{+}$T cell at sample level. r means the correlation coefficient calculated by Pearson's correlation test. $^{\#}p < 0.05$. Source data are provided as a Source Data file.

Supplementary Table 1. Age, sex and sample information for MTC and PTC patients in single-cell RNA sequence

Patient	Sex	Age	Lymph nodes metastasis	Distant Metastasis	Sample lesion	Cells
MTC01	Male	48	√	√	T	1976
MTC02	Famale	58	√		T	7397
					P	12059
					PBMC	7911
MTC03	Female	32			T	6894
					P	9653
					PBMC	8240
MTC04	Feamle	31	√	√	T	3354
					P	12199
					PBMC	13828
MTC05	Female	35	√		T	3327
					P	10364
					PBMC	9344
MTC06	Male	30	√		T	4244
					P	6052
MTC07	Female	52			T	5598
					P	5514
PTC01	Female	62	√		T	16171
					P	14266
					PBMC	8623
PTC02	Female	24	√		T	10900
					T2	6881
					P	5995
					PBMC	8623
PTC03	Female	39	√		T	10432
					P	7301
					PBMC	2788
PTC04	Female	45			T	8456
					T2	12406
					PBMC	7366
PTC05	Female	31	√		T	915
					P	923
					PBMC	826
PTC06	Female	37	√		T	7489
					P	7470
					PBMC	8580
PTC07	Female	72	√	√	T	10097
					P	6260
PTC08	Male	78	√		T	13407
