# Supplementary material for: The neurotransmitter calcitonin gene-related peptide shapes an immunosuppressive microenvironment in medullary thyroid cancer
Source: Nat Commun. 2024 Jul 19;15:5555. doi: 10.1038/s41467-024-49824-7 (PMC11271530; doi:10.1038/s41467-024-49824-7)
Supplement: Supplementary file 3 — Description of Additional Supplementary Files [file 41467_2024_49824_MOESM3_ESM.pdf]

Description of Additional Supplementary Files

Supplementary Data 1. Differentially expressed genes of major cell types.

Supplementary Data 2. The top 10 differentially expressed genes between PTC tumor cells and MTC tumor cells.

Supplementary Data 3. The top 10 differentially expressed genes of T cells.

Supplementary Data 4. The differentially expressed genes of $CD8^+$ T cells, $CD4^+$ T cells, NK and ILCs between PTC and MTC.

Supplementary Data 5. The clinical information of patients from sequencing in the study.

Supplementary Data 6. The gene signatures used in the study.

Supplementary Data 7. The clinical information of MTC patients in CGRP IHC analysis

Supplementary Data 8. Key resource table.
