# Supplementary material for: The neurotransmitter calcitonin gene-related peptide shapes an immunosuppressive microenvironment in medullary thyroid cancer
Source: Nat Commun. 2024 Jul 19;15:5555. doi: 10.1038/s41467-024-49824-7 (PMC11271530; doi:10.1038/s41467-024-49824-7)
Supplement: Supplementary file 12 — Reporting Summary [file 41467_2024_49824_MOESM12_ESM.pdf]

nature portfolio

Corresponding author(s): Haipeng Xiao, Yihao Liu

Last updated by author(s): Jun 14, 2024

# Reporting Summary

Nature Portfolio wishes to improve the reproducibility of the work that we publish. This form provides structure for consistency and transparency in reporting. For further information on Nature Portfolio policies, see our Editorial Policies and the Editorial Policy Checklist.

## Statistics

For all statistical analyses, confirm that the following items are present in the figure legend, table legend, main text, or Methods section.

| n/a | Confirmed | |
|---|---|---|
| [ ] | [x] | The exact sample size ($n$) for each experimental group/condition, given as a discrete number and unit of measurement |
| [ ] | [x] | A statement on whether measurements were taken from distinct samples or whether the same sample was measured repeatedly |
| [ ] | [x] | The statistical test(s) used AND whether they are one- or two-sided<br>*Only common tests should be described solely by name; describe more complex techniques in the Methods section.* |
| [x] | [ ] | A description of all covariates tested |
| [ ] | [x] | A description of any assumptions or corrections, such as tests of normality and adjustment for multiple comparisons |
| [ ] | [x] | A full description of the statistical parameters including central tendency (e.g. means) or other basic estimates (e.g. regression coefficient) AND variation (e.g. standard deviation) or associated estimates of uncertainty (e.g. confidence intervals) |
| [ ] | [x] | For null hypothesis testing, the test statistic (e.g. $F$, $t$, $r$) with confidence intervals, effect sizes, degrees of freedom and $P$ value noted<br>*Give P values as exact values whenever suitable.* |
| [x] | [ ] | For Bayesian analysis, information on the choice of priors and Markov chain Monte Carlo settings |
| [x] | [ ] | For hierarchical and complex designs, identification of the appropriate level for tests and full reporting of outcomes |
| [ ] | [x] | Estimates of effect sizes (e.g. Cohen's $d$, Pearson's $r$), indicating how they were calculated |

*Our web collection on statistics for biologists contains articles on many of the points above.*

## Software and code

Policy information about availability of computer code

| | |
|---|---|
| Data collection | All transcriptomic data reported in this study have been deposited at the Genome Sequence Archive at the National Genomics Data Center (Beijing, China) under the Accession ID HRA006084. |
| Data analysis | The software and R packages used in our study have been mentioned in Methods section. No unpublished software and algorithms are involved. |

For manuscripts utilizing custom algorithms or software that are central to the research but not yet described in published literature, software must be made available to editors and reviewers. We strongly encourage code deposition in a community repository (e.g. GitHub). See the Nature Portfolio guidelines for submitting code & software for further information.

## Data

Policy information about availability of data

All manuscripts must include a data availability statement. This statement should provide the following information, where applicable:

- Accession codes, unique identifiers, or web links for publicly available datasets
- A description of any restrictions on data availability
- For clinical datasets or third party data, please ensure that the statement adheres to our policy

The transcriptomic data reported in this study have been deposited at the Genome Sequence Archive at the National Genomics Data Center (Beijing, China) under the Accession ID HRA006084. Dataset HRA006084 is available under restricted access because of data privacy and supervision. For research purpose, access can be obtained by the DAC (Data Access Committees) of the GSA-human database. The approximate response time for accession requests is about one month. Once

access has been approved, the data will be available for two months. The remaining data are available within the Article, Supplementary Information or Source Data file. The single-cell publicly available data used in this study are available in the GEO database under accession code GSE163558[https://www.ncbi.nlm.nih.gov/geo/query/acc.cgi?acc=GSE163558], GSE176078[https://www.ncbi.nlm.nih.gov/geo/query/acc.cgi?acc=GSE176078], GSE151530[https://www.ncbi.nlm.nih.gov/geo/query/acc.cgi?acc=GSE151530], GSE148673[https://www.ncbi.nlm.nih.gov/geo/query/acc.cgi?acc=GSE148673], GSE193581[https://www.ncbi.nlm.nih.gov/geo/query/acc.cgi?acc=GSE193581], GSE191288[https://www.ncbi.nlm.nih.gov/geo/query/acc.cgi?acc=GSE191288], GSE148673[https://www.ncbi.nlm.nih.gov/geo/query/acc.cgi?acc=GSE148673], GSE217845[https://www.ncbi.nlm.nih.gov/geo/query/acc.cgi?acc=GSE217845], GSE223063[https://www.ncbi.nlm.nih.gov/geo/query/acc.cgi?acc=GSE223063], GSE181294[https://www.ncbi.nlm.nih.gov/geo/query/acc.cgi?acc=GSE181294]. The TCGA public transcriptome data used in our study are available in thyroid cancer part in UCSC xena dataset https://xenabrowser.net/datapages/.

## Research involving human participants, their data, or biological material

Policy information about studies with human participants or human data. See also policy information about sex, gender (identity/presentation), and sexual orientation and race, ethnicity and racism.

| | |
|---|---|
| Reporting on sex and gender | NA |
| Reporting on race, ethnicity, or other socially relevant groupings | NA |
| Population characteristics | All patients demographics are shown in Supplementary Data 5 and 7. |
| Recruitment | The recruitment process was unbiased. |
| Ethics oversight | The study was approved by the Institutional Research Ethics Committee of The First Affilated Hospital of Sun Yat-sen University ([2021]109). The informed consents were collected from all patients for their willingness to participate in our project. |

Note that full information on the approval of the study protocol must also be provided in the manuscript.

# Field-specific reporting

Please select the one below that is the best fit for your research. If you are not sure, read the appropriate sections before making your selection.

[x] Life sciences  [ ] Behavioural & social sciences  [ ] Ecological, evolutionary & environmental sciences

For a reference copy of the document with all sections, see nature.com/documents/nr-reporting-summary-flat.pdf

# Life sciences study design

All studies must disclose on these points even when the disclosure is negative.

| | |
|---|---|
| Sample size | This was a biological study instead of a clinical trial, so we did not conduct a hypothesis test for sample size calculation. In this study, we included all available MTC samples for single-cell RNA analysis during 2021-2023 in our hospital. A total of 39 tissue and PBMC samples from 15 patients were used for single-cell RNA analysis, and 16 samples from 8 patients were used for Bulk-RNA analysis. Additionally, 181 samples from 181 patients were used for multiplex immunohistochemistry or immunohistochemistry assays as validation cohort. |
| Data exclusions | No data was excluded from the analysis. |
| Replication | The IHC assays were largely replicated in Fig. 1G and mIHC assays were performed in 18 different patients. The in vitro experiments were repeated three times. |
| Randomization | NA |
| Blinding | NA |

# Reporting for specific materials, systems and methods

We require information from authors about some types of materials, experimental systems and methods used in many studies. Here, indicate whether each material, system or method listed is relevant to your study. If you are not sure if a list item applies to your research, read the appropriate section before selecting a response.

## Materials & experimental systems

| n/a | Involved in the study |
|---|---|
| ☐ | ☒ Antibodies |
| ☒ | ☐ Eukaryotic cell lines |
| ☒ | ☐ Palaeontology and archaeology |
| ☒ | ☐ Animals and other organisms |
| ☒ | ☐ Clinical data |
| ☒ | ☐ Dual use research of concern |
| ☒ | ☐ Plants |

## Methods

| n/a | Involved in the study |
|---|---|
| ☒ | ☐ ChIP-seq |
| ☐ | ☒ Flow cytometry |
| ☒ | ☐ MRI-based neuroimaging |

# Antibodies

| | |
|---|---|
| Antibodies used | All antibodies used in this study are described in details in Methods section and Supplementary Data 8. |
| Validation | Immunohistochemistry antibodies were commercially obtained and sources are listed in Methods section. Antibodies were validated based on control tissue samples and pathologist review. |

# Plants

| | |
|---|---|
| Seed stocks | NA |
| Novel plant genotypes | NA |
| Authentication | NA |

# Flow Cytometry

## Plots

Confirm that:

☒ The axis labels state the marker and fluorochrome used (e.g. CD4-FITC).

☒ The axis scales are clearly visible. Include numbers along axes only for bottom left plot of group (a 'group' is an analysis of identical markers).

☒ All plots are contour plots with outliers or pseudocolor plots.

☒ A numerical value for number of cells or percentage (with statistics) is provided.

## Methodology

| | |
|---|---|
| Sample preparation | Sample preparation process and detailing resource were described in "Monocyte isolation and DC induction" and "Flow cytometry" parts of Methods. |
| Instrument | CYTEK Aurora flow cytometry |
| Software | Data was collected by complementary software of CYTEK Aurora flow cytometry and analyzed by Flow Jo v10.6.2. |
| Cell population abundance | The abundance of cultured DCs in collected cells is 85-95%. |
| Gating strategy | First, we used FSC-H/SSC-H gate to identify lymphcytes. Next, we used zombie-violet to identify live cells. Dendritic cells were confirmed by both CD45 and CD11c positive and the boundaries were defined by unstained cells. |

☒ Tick this box to confirm that a figure exemplifying the gating strategy is provided in the Supplementary Information.
